# Supplementary material for: Non-Markovian cost function for quantum error mitigation with Dirac Gamma matrices representation
Source: Sci Rep. 2023 Nov 16;13:20069. doi: 10.1038/s41598-023-45053-y (PMC10654775; doi:10.1038/s41598-023-45053-y)
Supplement: Supplementary file 1 — Supplementary Information. [file 41598_2023_45053_MOESM1_ESM.docx]

**Supplementary Information for**

**Non-Markovian cost function for quantum error mitigation with Dirac Gamma matrices representation**

Doyeol Ahn^1,*^

^1^Department of Electrical and Computer Engineering,

University of Seoul, 163 Seoulsiripdae-ro, Tongdaimoon-gu, Seoul 02504, Republic of Korea

*Corresponding author: [dahn@uos.ac.kr](mailto:dahn@uos.ac.kr)

**I. Time-convolutionless evolution of a non-Markovian reduced-density operator**

The total Hamiltonian for an open two-state system is given by [1-5]


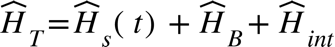
 (1)

where
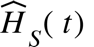
is the system Hamiltonian for a two-state system,
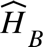
 the Hamiltonian acting on the reservoir or an environment,
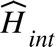
 is the interaction between the system and the environment (as depicted in figure 1).

The equation of motion for the total density operator $\rho_{T}(t)$ of the total system is given by a quantum Liouville equation [1]


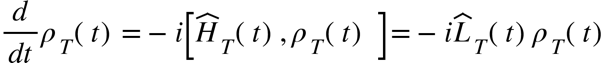
, (2)

where


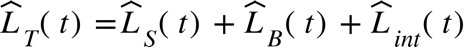
 (3)

is the Liouville super operator in one-to-one correspondence with the Hamiltonian. Here, we use the unit in which . In order to derive an equation and to solve for a system alone, it is convenient to use the projection operators which decompose the total system by eliminating the degrees of freedom for the reservoir. We define thine-independent projection operator $\underline{P}$ and $\underline{Q}$ given by [1,2]


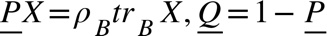
 (4)

for any dynamical variable $X$. Here
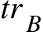
 denotes a partial trace over the quantum reservoir and
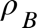
 is the density matrix of the reservoir. The projection operators satisfy the following operator identities:


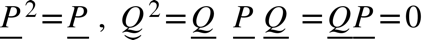
, (5a)


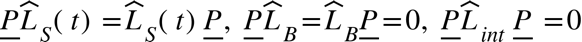
, (5b)

and
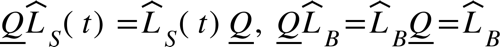
. (5c)

The information of the system is then contained in the reduced density operator


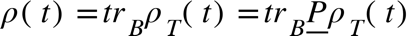
. (6)

If we multiply equation (2) by$\underline{P}$and$\underline{Q}$ on the left side, we obtain coupled equations for $\underline{P}\rho_{T}(t)$ and $\underline{Q}\rho_{T}(t)$ as follows:


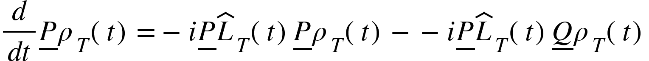
, (7a)

and


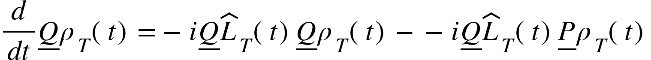
, (7b)

where we have modified the Eq. (2) as


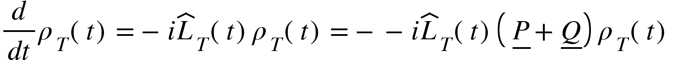
.

We assume that the system was turned on at $t=0$ and the input state prepared at $t=0$ was isolated with the reservoir such that
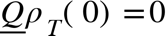
. The formal solution of (7b) is given by


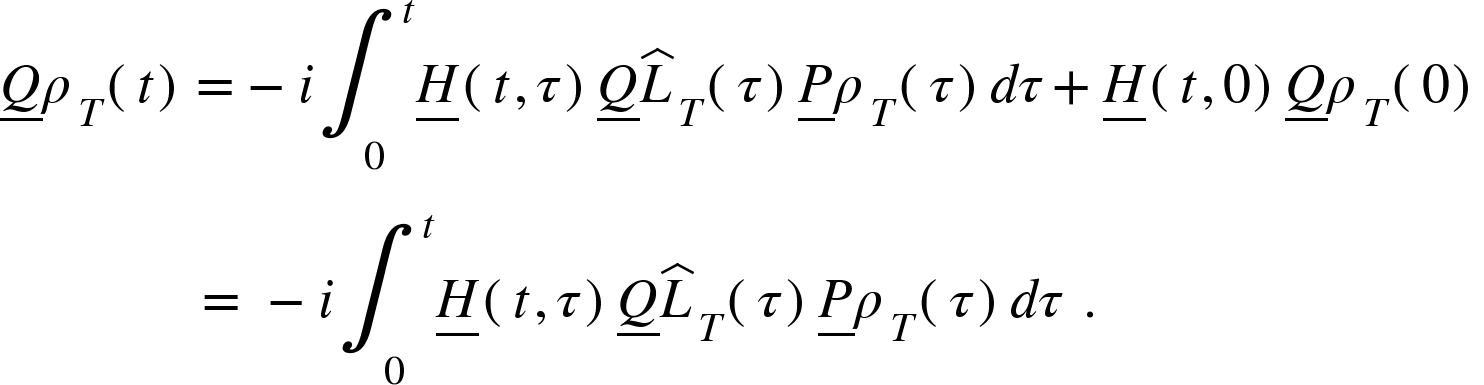
 (8)

where the projected propagator
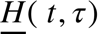
 of the total system is defined by


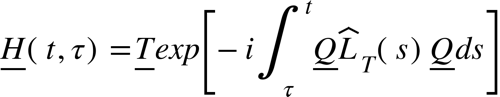
. (9)

Here $\underline{T}$ is the time-ordering operator. We also introduce an anti-time evolution operator $\underline{G}(t,\tau)$ which is defined by


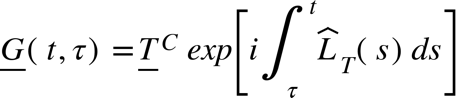
 , (10)

such that
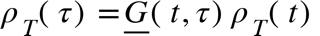
 . (11)

It is not very difficult to show [1] that


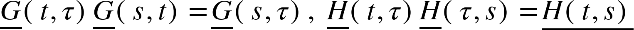
. (12)

Here
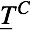
 is anti-time-ordering operator.

From Eqs. (8)-(11), we obtain


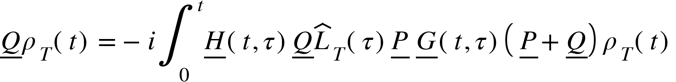
 (13)

which is obviously in time-convolutionless form. Eq. (13) can be rewritten as


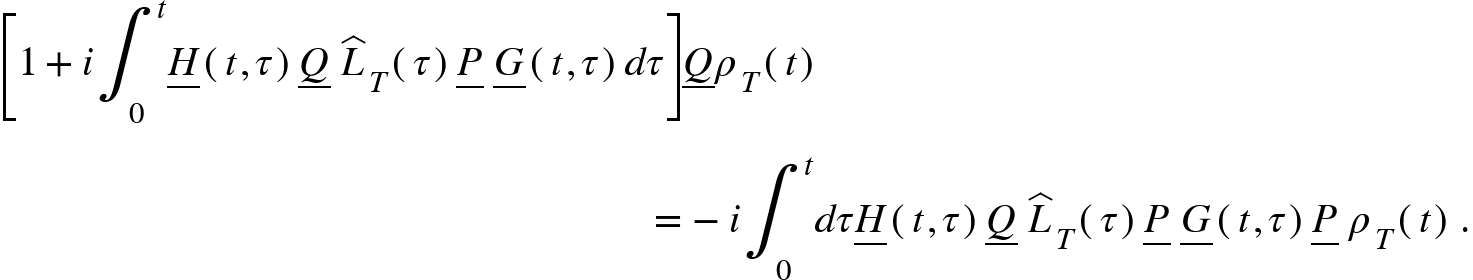
 (14)

We introduce new super operators $\hat{g}(t)$ and $\hat{\theta}(t)$ which are given by


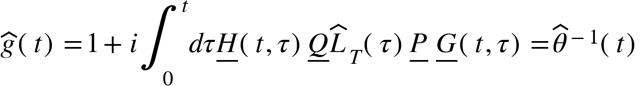
. (15)

Then from Eqs. (13)-(15), we obtain


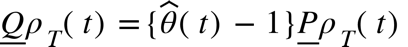
 (16)

and


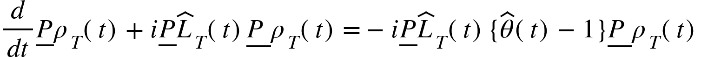
. (17)

After some mathematical manipulations, we obtain the formal solution of Eq. (17), which is given by


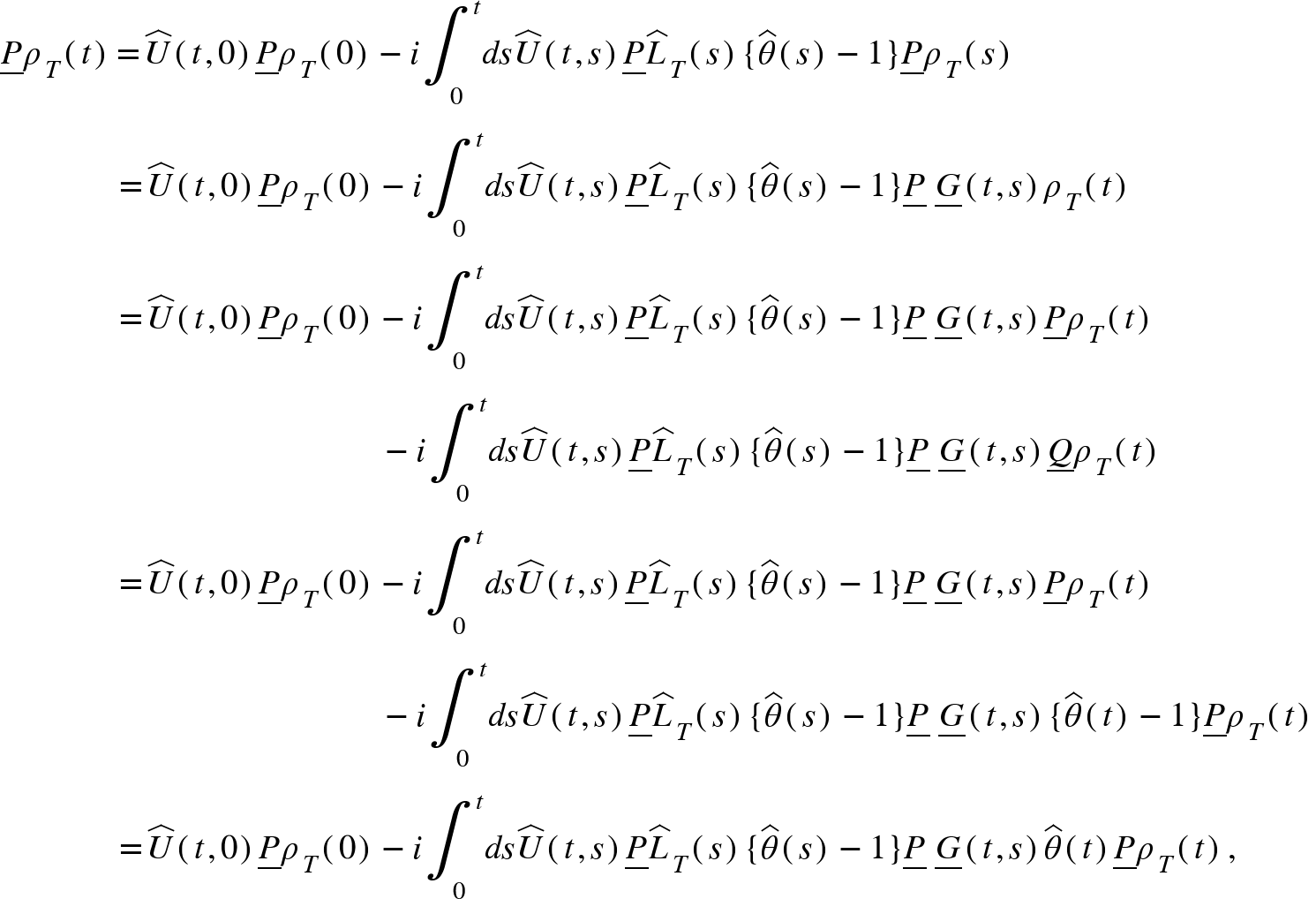
 (18)

where


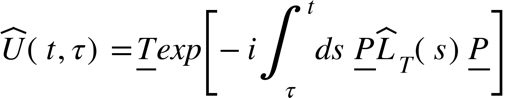
 (19)

is the projected propagator of the total system.

From,
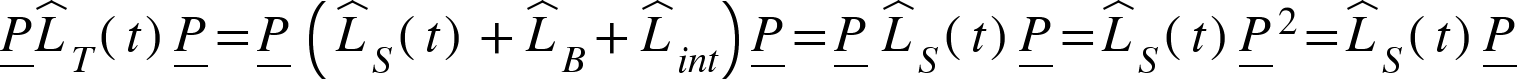
 , we obtain


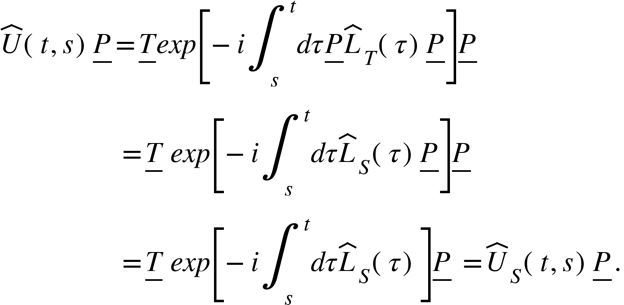
 (20)

Here
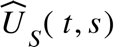
is the propagator of the system.

From Eqs. (4) and (6), we get


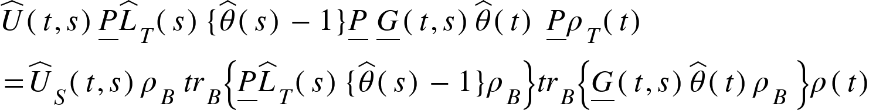


By the way, by substituting Eq. (7) into Eqs. (18)-(20), we obtain


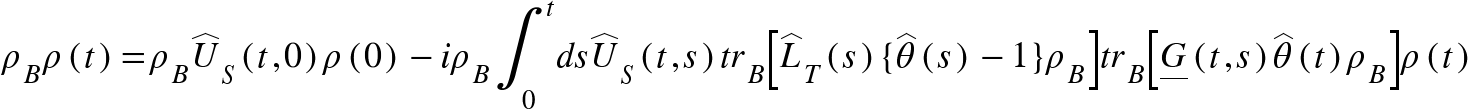
,

(21)

or


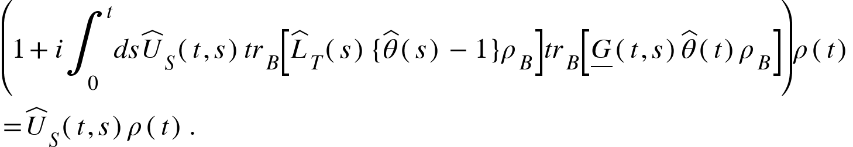
 (22)

If we define
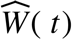
by


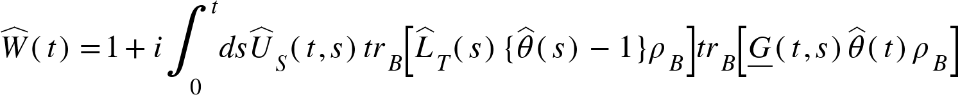
, (23)

Then, the evolution operator for the reduced density operator
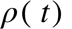
is given by


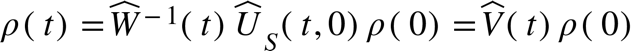
, (24)

where the super-operator
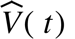
for the evolution of the reduced density operator is defined by
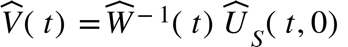
. Within the Born approximation, we have


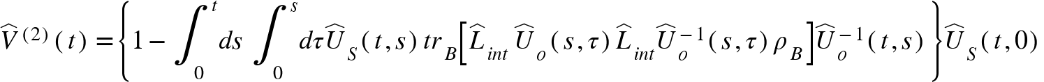
, (25)

where


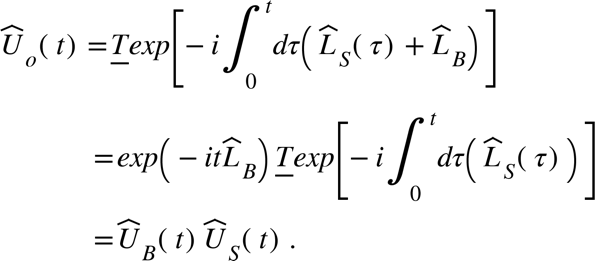
 (26)

A detailed derivation of Eq. (25) is given in the below:

We start with Eq. (23)


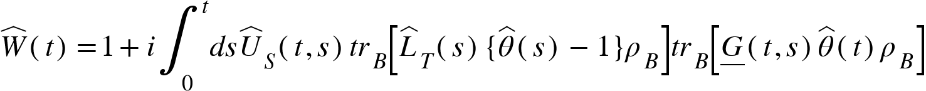
. (23)

We define


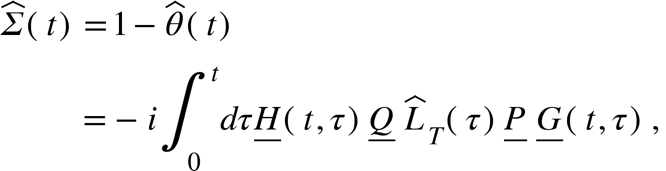
 (27)

then


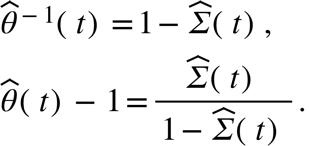
 (28)

Also, we have


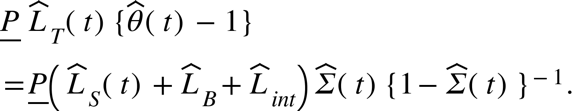


The detailed expression for
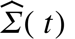
becomes


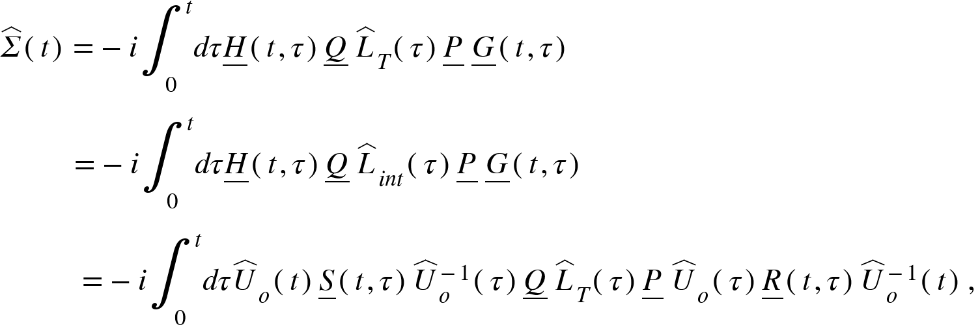
 (29)

where


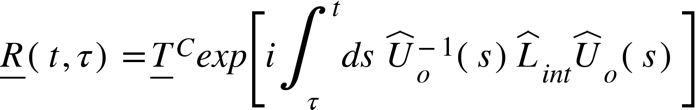
 (30)

is the evolution operator in the interaction picture and


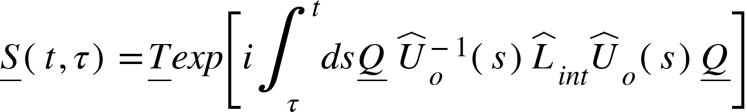
 (31)

is the projected propagator of the total system in the interaction picture. Then Eq. (23) can be rewritten as


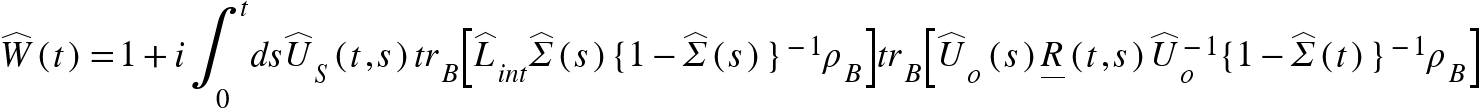
.

(32)

where


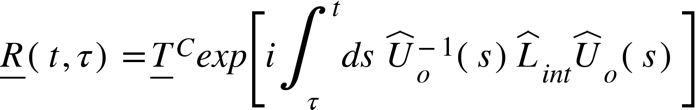
 (33)

is the evolution operator in the interaction picture and


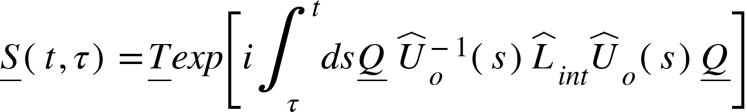
 (34)

is the projected propagator of the total system in the interaction picture. Then Eq. (23) can be rewritten as


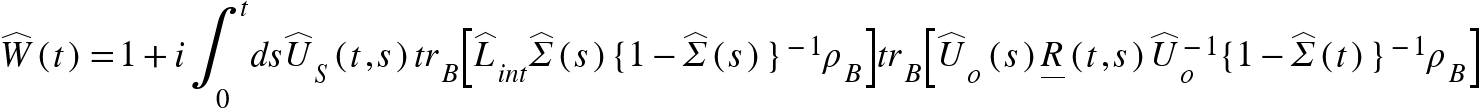
.

(35)

Born approximation of Eq. (35) leads to


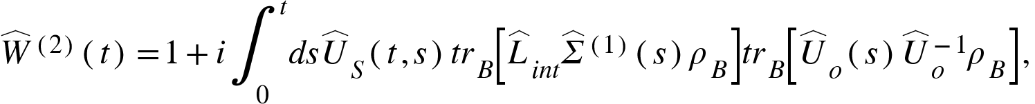
 (36)

where


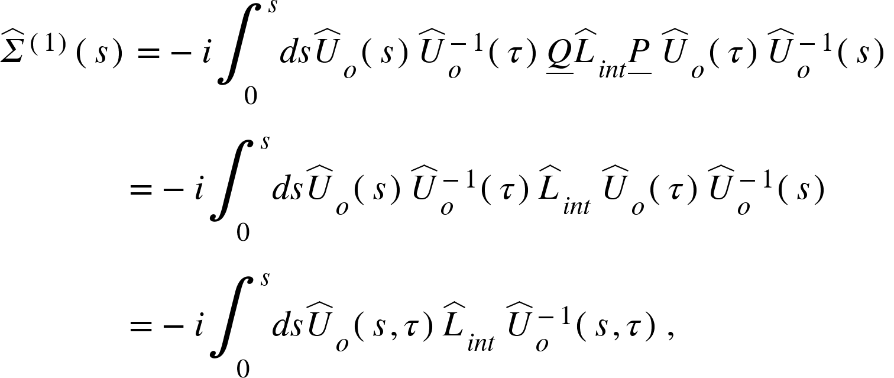
 (37)

using the ansatz
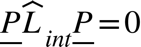
. From Eqs. (36) and (37), we get


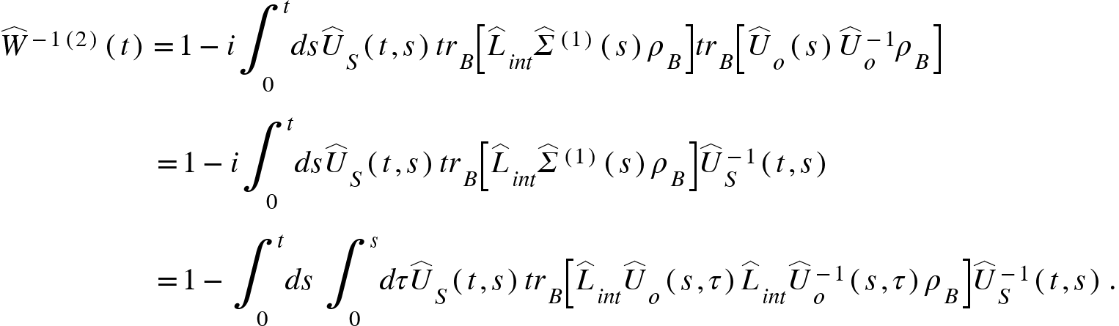
(38)

By substituting Eq. (38) into
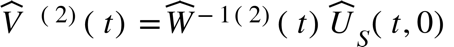
, we obtain Eq. (25).

After some mathematical manipulations,
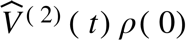
becomes
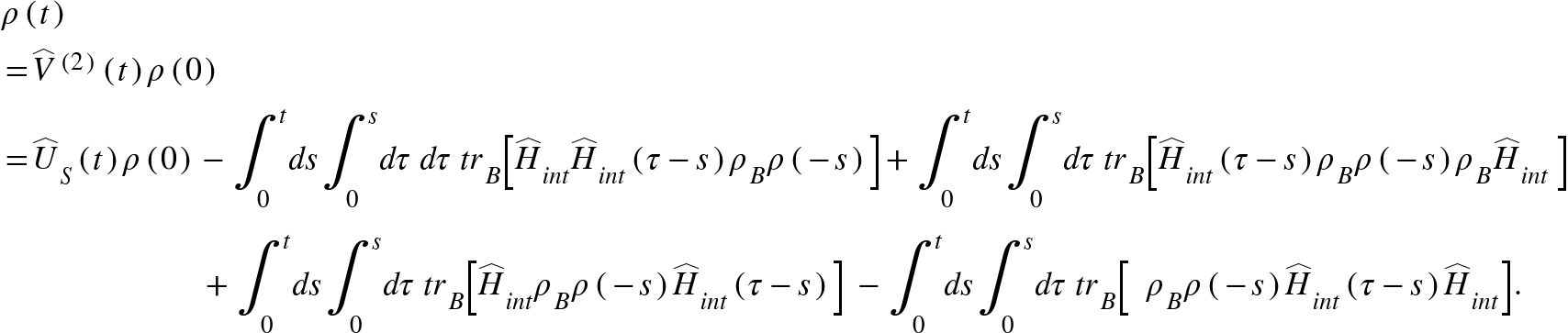
 (39)

Here
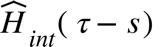
and
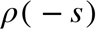
are Heisenberg operators defined by


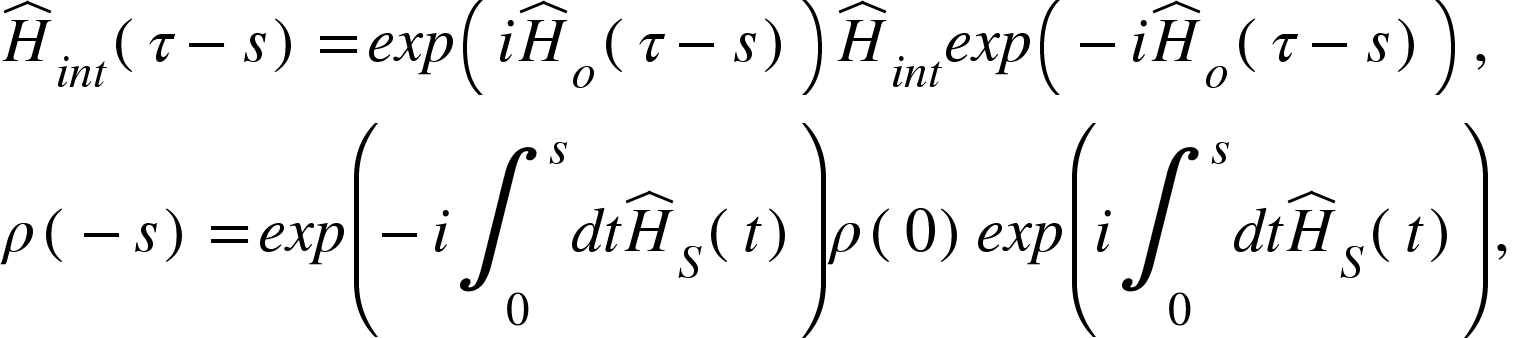


respectively.

**Non-Markovian evolution of two-qubit gate operation**

In this work, we focus on the two-qubit gate operations and model the interaction of the quantum system with the environment during the gate operation by a Caldeira-Leggett model [3-5] where a set of harmonic oscillators are coupled linearly with the system spin by


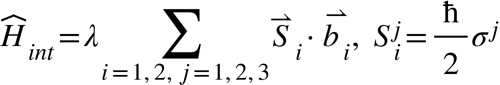
 (40)

where
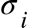
 is the Pauli matrices
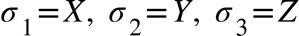
 and
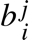
 is the fluctuating quantum field associated the i^th^ qubit, whose motion is governed by the harmonic-oscillator Hamiltonian.

In the evaluation of Eq. (39), we obtain the following relations [1]:


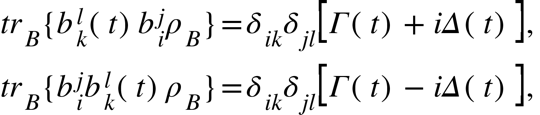
 (41)

where


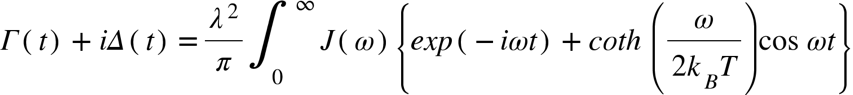
. (42)

Here
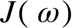
is the ohmic damping given by
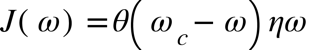
,
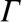
 is the decoherence rate of the qubit system.

We evaluate the reduced-density-operator in the multiplet basis representation [1]


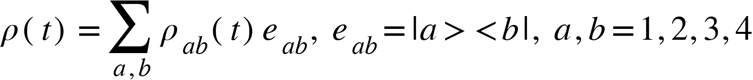
, (43)

where
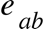
 is the multiplet states. The inner product between the multiplet basis is defined by


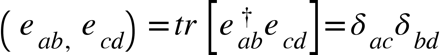
. (44)

Then, from Eqs. (39)-(44), we obtain the matrix component of the reduced-density-operator as


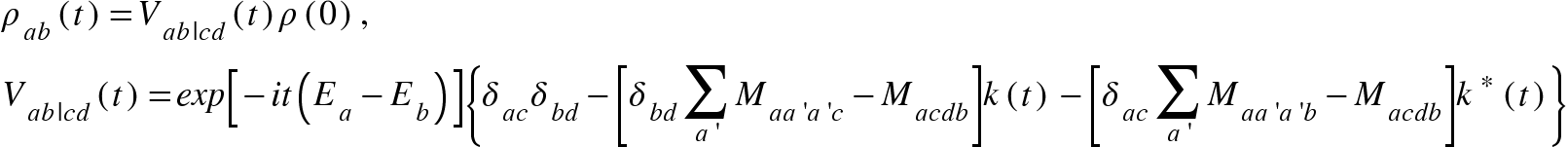
 (45)

where


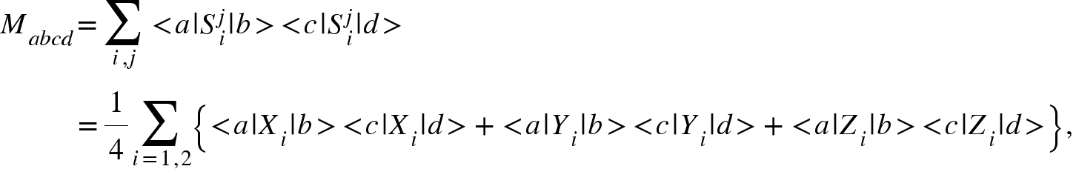
 (46)

and
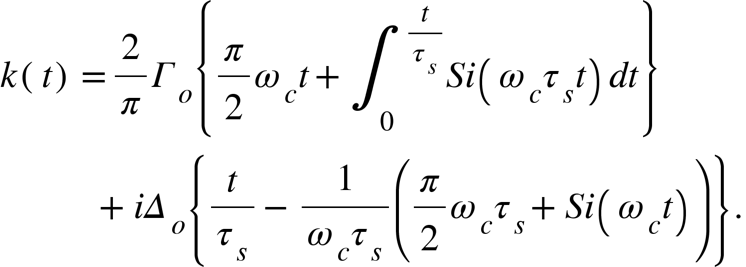
 (47)

Here
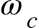
 is the high frequency cutoff,
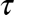
is the switching time,
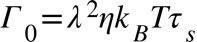
 and
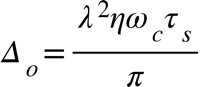
.

We now study the non-Markovian errors associated with two-qubit gate operations. Here,
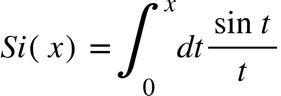
, is a sine integral. For reference, we also note the cosine integral,
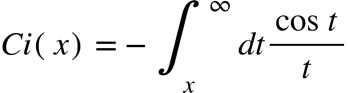
.

Derivation of Equations (45) to (47) is given in the below:

We define the operator integrand
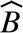
 as follows:


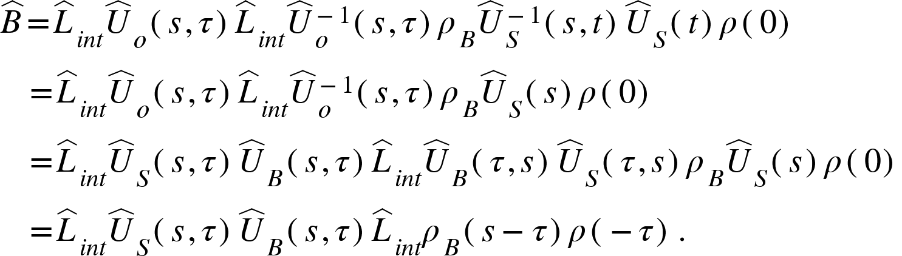
 (48)

Here, we used the relation


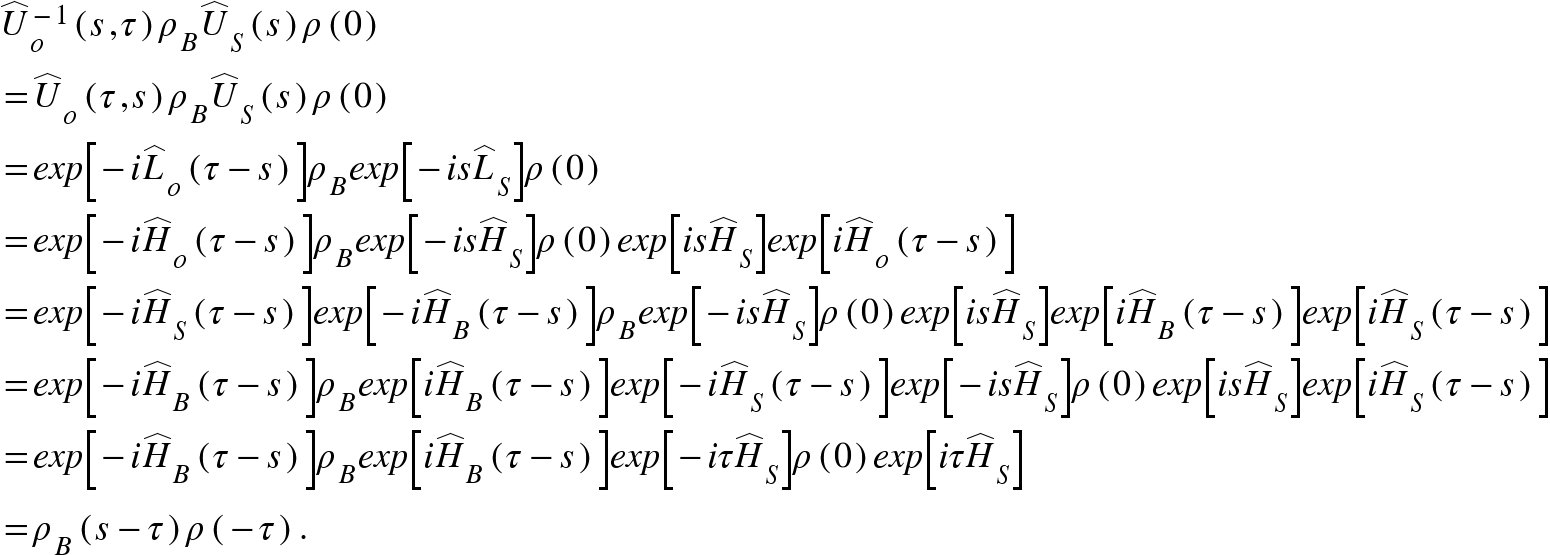


(49)

In Eq. (49), we have used Baker-Campbell-Hausdorff formula


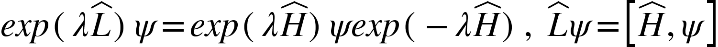
. (50)

Equation (48) is further expanded as


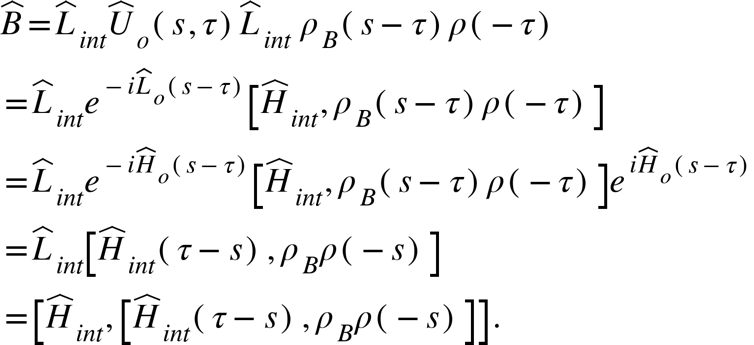
 (51)

Substituting Eq. (40) and take a trace over the environment, we get


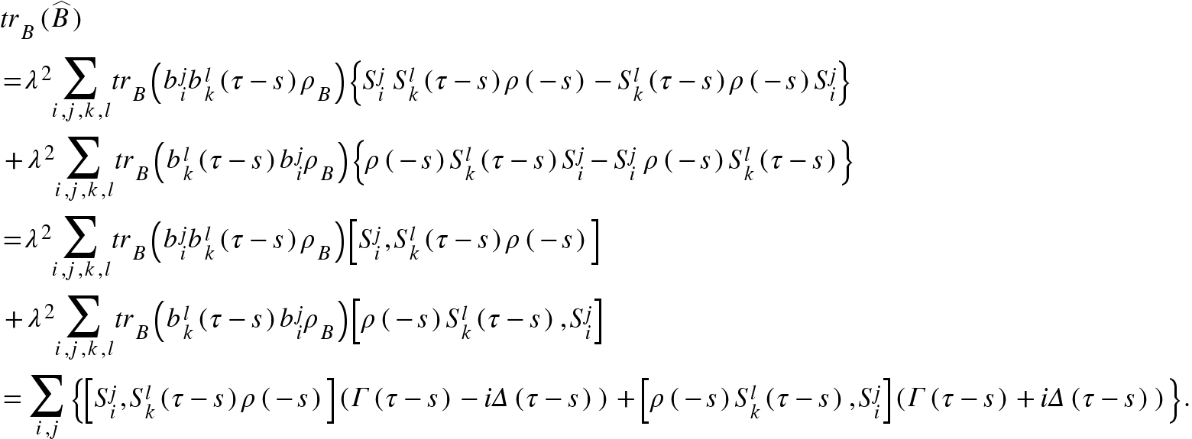
 (52)

Then, we obtain


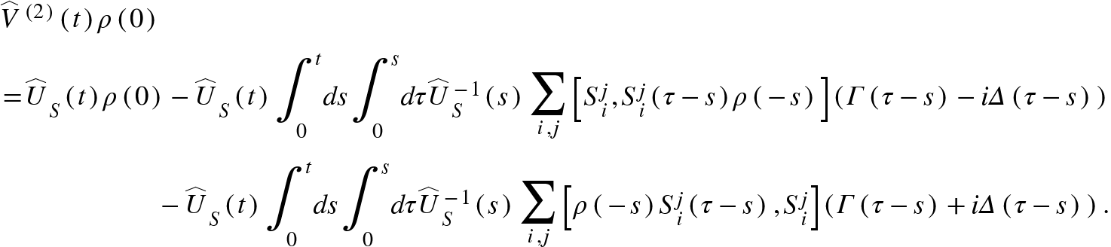
 (53)

If we evaluate
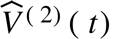
in the multiplet basis, we obtain


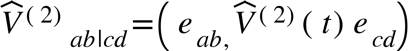
. (54)

Let’s define operator
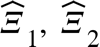
 as follows:


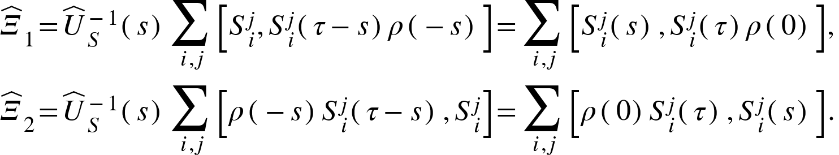


We first calculate
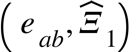
which becomes

(55)

Likewise, becomes

(55)

where

(56)

We define as

(57)

By integrating Eq. (57) over , we obtain

(58)

Equation (42) becomes

(59)

In order to evaluate the integrals in (58) and (59), we need to calculate the integral , which is given by

(60)

In Eq. (60), we have used the following relations [6]:

(61)

We now define , which is given by

(62)

From Eqs. (58)-(62), we obtain given by

(63)

This establishes Eqs. (45) to Eq. (47).

**III. Two-qubit gate operations with non-Markovian noise sources**

**SWAP operation**

We first consider the SWAP gate operation for various input states.

We now consider the non-Markovian error associated with SWAP gate operation. For SWAP gate operation, the multiplet basis is given by

(64)

In NISQ machines, the input and out states are represented by the computational basis:

. (65)

Then the reduce-density-operator in the computational basis is given by

(66)

Case 1: When that the initial state is given by , we obtain

(67)

Using Eqs. (64)-(66) and (67), we obtain the computational basis representation of the reduced-density-operator as

(68)

Case 2: When that the initial state is given by , we obtain

(69)

Using Eqs. (64)-(67) and (69), we obtain the computational basis representation of the reduced-density-operator as

(70)

Case 3: When that the initial state is given by , we obtain

(71)

Using Eqs. (64)-(67) and (71), we obtain the computational basis representation of the reduced-density-operator as

(72)

Case 4: When that the initial state is given by , we obtain

(73)

Using Eqs. (66)-(67) and (73), we obtain the computational basis representation of the reduced-density-operator as

(74)

**Identity operation**

We now consider the non-Markovian error associated with Identity gate operation. For Identity gate operation, the multiplet basis is the same as the computational basis given by

(75)

Case 1: When that the initial state is given by , we obtain

(76)

Case 2: When that the initial state is given by , we obtain

(77)

Case 3: When that the initial state is given by , we obtain

(78)

Case 4: When that the initial state is given by , we obtain

(79)

We have tried both ibm_guadalupe through IBM Quantum and IonQ through Amazon Braket to compare the theory with the experiment for the non-Markovian errors associated with SWAP and Identity operations.

1. 1. Ahn, D., Oh, J. H., Kimm, K. & Hwang, S, W. *Time-convolutionless reduced-density-operator theory of a noisy quantum channel: two-bit quantum gate for quantum-information processing*. Phys. Rev. A **61**, 052310 (2000).
2. 2. Ahn, D. Lee, J., Kim, M. S. & S. W. Hwang, *Self-consistent non-Markovian theory of a quantum state evolution for quantum information processing*. Phys. Rev. **A** 66, 012302 (2002).
3. 3. Hall, M. J. W. Canonical form of master equations and characterization of non-Markovianity. Phys. Rev. A 89, 042120 (2014).
4. 4. De Vega, I. & Alonso, D. Dynamics of non-Markovian quantum systems. Rev. Mod. Phys. 89, 015001 (2017).
5. 5. Loss, D. & DiVincenzo, D. P. Quantum computation with quantum dots, Phys. Rev. A 57, 120 (1998).
6. 6. Gradshteyn, I. S. & Ryzhik, I. M. *Table of Integrals, Series and Products*. (Academic Press, San Diego, 2007)
